# Collaborative pedagogies in primary physical education: a comparative analysis of tandem teaching models in North Macedonia and Slovakia

**DOI:** 10.3389/fspor.2026.1754410

**Published:** 2026-03-04

**Authors:** Gabriela Luptáková, Biljana Popeska, Hristina Ristevska, Tibor Balga, Ilija Klincarov, Branislav Antala

**Affiliations:** 1 Faculty of Physical Education and Sport, Comenius University in Bratislava, Bratislava, Slovakia; 2 Faculty of Educational Sciences, Goce Delcev University, Stip, North Macedonia; 3 Municipal Secondary School “Taki Daskalo”, Bitola, North Macedonia; 4 Faculty of Physical Education, Sport and Health, Saints Cyril and Methodius University of Skopje, Skopje, North Macedonia

**Keywords:** benefits, challenges, generalist teacher, physical education, physical education teacher, primary school, sports coach, tandem teaching

## Abstract

This study rigorously compares the perceptions and operational differences of generalist teachers within two distinct national tandem teaching models in primary Physical Education (PE): the rotational sports coach-generalist teacher model in Slovakia and the sustained PE specialist-generalist teacher model in North Macedonia. The research examines implementation quality, systematic support, task division, benefits, and challenges. A questionnaire was completed by 618 generalist teachers from Slovakia (*n* = 314) and North Macedonia (*n* = 304). This comparative study utilized a cross-sectional design with a self-administered questionnaire, employing descriptive statistics, t-tests for mean comparisons, and Chi-square tests with Cramér's V to quantify effect sizes for categorical associations. Both countries exhibited a similarly high overall positive perception of the tandem concept (*p* = 0.240). However, North Macedonian teachers reported significantly better systemic support (*p* ≤ 0.012) and highly significant differences in role division (*p* < 0.001). Large effect sizes were specifically found in communication with parents (V = 0.540), while moderate to strong effects were observed for lesson planning (V = 0.495) and student preparation (V = 0.417). Key findings indicate that the North Macedonian model fosters a more collaborative environment with shared responsibility, whereas the Slovak model tends toward vertical specialization and delegated tasks. Additionally, resource constraints—specifically the lack of adequate space and equipment—remain the most significant shared barrier to effective implementation in both national contexts. To optimize collaborative teaching, policy recommendations should incorporate North Macedonia's tandem structure (sustained collaboration with an internal PE specialist) and systematic support, along with Slovakia's clarity in administrative delegation, reinforced by mandated infrastructure investment and dedicated co-planning time.

## 1 Introduction

To provide quality education, the growing complexity of modern schooling has amplified the demand for collaborative pedagogies, the effectiveness of which significantly affects the provision of quality PE. While co-teaching and team teaching are broad terms for various collaborative models, tandem teaching in this study refers to a specific, structured partnership between a generalist teacher and a PE specialist (or coach) designed to bridge the gap between general pedagogical skills and subject specific expertise.

Defined by “*two or more professionals delivering substantive instruction to a diverse, or blended, group of students in a single physical space”* [(1), p. 1], its effectiveness is rooted in a continuous cycle of co-planning, co-teaching, co-assessing, and co-reflecting (2, 3). This approach has shown significant pedagogical benefits for students, creating a richer learning environment characterized by diverse perspectives (4) and providing enhanced support that allows teachers to more effectively address the needs of diverse learners (5, 6). For the educators themselves, tandem teaching offers valuable opportunities for professional growth and personal development, significantly enhancing pedagogical skills and self-confidence (7, 8). The specific instructional practices employed by co-teachers determine their definitive effect (9), prompting extensive literature to categorize various forms of collaboration, such as the six core models delineated by Friend et al. (10). In this study, we operate under a conceptual model where the policy structure (e.g., mandatory vs. voluntary participation) serves as the primary driver that dictates the formal division of roles and the level of systemic support provided to educators. These structural factors are theorized to be highly interrelated, as they collectively shape teachers’ perceptions of benefits and challenges, ultimately determining the perceived quality of the collaborative implementation. However, effective implementation is frequently challenged by systemic barriers, including insufficient dedicated planning time (11), the prevalence of asymmetrical power dynamics where one teacher assumes a subordinate role (12), and a widespread deficit in formal professional training in collaborative practices (13–15). A key constraint is that frequent changes in teacher pairings limit the development of consistency, thereby undermining collaborative instructional practices (16, 17).

The application of tandem teaching has become increasingly relevant in primary PE, often in response to persistent concerns that generalist classroom teachers may not consistently deliver quality PE at the required standard [e.g., (18–21)]. In the PE context specifically, research suggests this model can confer unique advantages, such as increasing student physical activity both during lessons and across the school day (22). This approach has been observed globally, encompassing models featuring external sports coaches in contexts like the UK (20, 23), Ireland (24), or Slovakia [e.g., (25), and collaboration with internal PE teachers in North Macedonia (22, 26). These varying national applications present two principal tandem approaches—internal and external, as identified by Antala (27)]. Two national programs—Slovakia and North Macedonia—provide a unique, comparative case study. Slovakia utilizes the external coach model, pairing generalist teachers with external sports coaches in a voluntary structure. A key feature of this program is the coaching rotation model, where a new coach is assigned every ten weeks, and coaches collaborate for only one of the three mandated weekly PE lessons (25, 28–30). In stark contrast, North Macedonia utilizes a state-level internal PE teacher model, where collaboration is mandatory and utilized for all weekly PE lessons. The PE teachers maintain continuity by remaining assigned to the same students and generalist teachers for at least one full academic year (26, 29). In evaluating these models, it is critical to acknowledge a potential source of bias in the comparative design: the mandatory, legislated nature of participation in North Macedonia compared to the voluntary, initiative-based participation in Slovakia. This difference in legal and systemic commitment may naturally influence teacher motivation and their subsequent reporting on implementation quality and role involvement.

While the foundational principles of tandem teaching are well-documented, a significant gap remains in the detailed comparative analysis of successful collaboration and actual good practices across distinct educational professions in different systemic contexts (31).

Moving beyond the general identification of obstacles in collaborative teaching (32), this research addresses the critical need for studies offering concrete, evidence-based guidance for practitioners (33). This is achieved through a rigorous comparative analysis of generalist teachers' perceptions and experiences within two distinct national tandem teaching models in PE. Specifically, the study aims to: (1) Compare the functional involvement and division of responsibilities among generalist teachers in key pedagogical and organizational tasks; (2) Evaluate and contrast their overall perception of tandem teaching implementation quality; and (3) Identify and compare the primary benefits and challenges encountered within each model. Furthermore, the research examines and contrasts generalist teachers' agreement with statements regarding systemic and programmatic support and analyzes their prescriptive suggestions for necessary policy and logistical improvements. The ultimate goal is to inform evidence-based policy and practice recommendations by highlighting the unique strengths and critical areas for improvement inherent in each national model, thereby optimizing collaborative teaching strategies in primary PE.

## 2 Materials and methods

### 2.1 Study participants and recruitment

The participant cohort for this investigation consisted of in-service generalist classroom teachers drawn from the public school systems of Slovakia and North Macedonia. A primary prerequisite for inclusion was documented evidence of a minimum of one year of experience engaging in tandem teaching within the context of PE. The study collected data from a total of 618 in-service generalist teachers who completed the self-administered questionnaire. The resulting sample was closely divided between the two nations: 314 from Slovakia (50.8%) and 304 from North Macedonia (49.2%).

Pertaining to gender, the cohort was predominantly female, accounting for 83.3% (*n* = 515) of the total sample. Cross-national analysis of the gender distribution revealed a notable asymmetry (Table 1): the Slovakian sub-sample was overwhelmingly female, while the North Macedonian sub-sample, although still female-dominant, exhibited a less pronounced distribution.

**Table 1:** Distribution of study participants by country and gender.

Variable	Country		Total	
	Slovakia	North Macedonia		
Gender	Male	10	93	103
	Female	304	211	515
Total	314	304	618	

The participants’ professional experience was broadly distributed across several categories (see Table 2). The largest combined segments of the cohort reported either 11–20 years (25.7%) or up to 5 years (24.4%) of service. Notably, the distribution demonstrated pronounced cross-national variation: the Slovakian sub-sample was primarily composed of educators with more than 30 years of experience (32.8%). This contrasts sharply with the North Macedonian group, which showed high concentrations in the up to 5-year and 11–20-year ranges (29.9% each). Furthermore, the 5–10-year experience range was unrepresented in the Slovakian participants.

**Table 2:** Working experience of study participants by country.

Working Experience	Slovakia (Count & %)	North Macedonia (Count & %)	Total (Count & %)
Until 5 years	60 (19.1%)	91 (29.9%)	151 (24.4%)
5 to 10 years	0 (0%)	48 (15.8%)	48 (7.8%)
11 to 20 years	68 (21.7%)	91 (29.9%)	159 (25.7%)
21 to 30 years	83 (26.4%)	52 (17.1%)	135 (21.8%)
More than 30 years	103 (32.8%)	22 (7.2%)	125 (20.2%)
Total	314 (100%)	304 (100%)	618 (100%)

Experience in tandem teaching was most commonly reported as one year across the total sample (28.0%). This initial experience level was heavily skewed toward Slovakian participants, who constituted a significant majority of the one-year group. Conversely, longer tenures of four and five years of tandem experience were markedly more prevalent in the North Macedonian sub-sample, suggesting a more sustained practice in that national context (see Table 3).

**Table 3:** Tandem experience of study participants by country.

Tandem Experience	Slovakia (Count & %)	North Macedonia (Count & %)	Total (Count & %)
1 year	134 (42.7%)	39 (12.8%)	173 (28%)
2 years	94 (29.9%)	54 (17.8%)	148 (23.9%)
3 years	61 (19.4%)	66 (21.7%)	127 (20.6%)
4 years	16 (5.1%)	48 (15.8%)	64 (10.4%)
5 years	9 (2.9%)	97 (31.9%)	106 (17.2%)
Total	314 (100%)	304 (100%)	618 (100%)

Regarding grade-level involvement, the largest segment of the cohort taught at the first grade level (32.7%) (see Table 4). The distribution showed a clear cross-national divergence: Slovakian teachers were predominantly involved in the first and second grades, whereas the largest proportion of North Macedonian teachers was engaged in multi-grade teaching.

**Table 4:** Grade-Level involvement of study participants by country.

Grade	Slovakia (Count & %)	North Macedonia (Count & %)	Total (Count & %)
First Grade	128 (40.8%)	74 (24.3%)	202 (32.7%)
Second Grade	95 (30.3%)	47 (15.5%)	142 (23.0%)
Third Grade	46 (14.6%)	8 (2.6%)	54 (8.7%)
Fourth Grade	20 (6.4%)	55 (18.1%)	75 (12.1%)
Fifth Grade	0 (0%)	34 (11.2%)	34 (5.5%)
Multi-Grade Teaching	25 (8.0%)	86 (28.3%)	111 (18.0%)
Total	314 (100%)	304 (100%)	618 (100%)

A substantial proportion of the sample voluntarily disclosed their teaching location (Slovakia: 27.1%; North Macedonia: 29.3%). This self-reported data demonstrated excellent geographic diversity, encompassing all eight administrative regions of both Slovakia and North Macedonia (see Table 5). This comprehensive geographical coverage suggests a robust degree of regional representativeness of the respective generalist teacher populations and indicates the extensive establishment of the tandem teaching project across both nations.

**Table 5:** Regional representation of study participants in Slovakia and north Macedonia.

Slovakia			North Macedonia		
Region	Teachers (*n* = 85)	Percentage (%)	Region	Teachers (*n* = 89)	Percentage (%)
Košice region	19	22.35	Vardar region	14	15.73
Žilina region	15	17.65	East Region	11	12.36
Bratislava region	12	14.12	South—west region	9	10.11
Prešov region	11	12.94	South—east region	12	13.48
Banská Bystrica region	10	11.76	Pelagonia region	12	13.48
Nitra region	7	8.24	Polog region	5	5.62
Trenčín region	6	7.06	North—east region	7	7.87
Trnava region	5	5.88	Skopje region	19	21.35

Recruitment was managed through distinct national mechanisms. In Slovakia, teacher participation was facilitated via the national initiative, “Coaches in Schools,” which had established the generalist teacher-sports coach tandem. Potential participants were identified and contacted using email addresses provided by school representatives, overseen by a program coordinator from the National Institute of Education and Youth (an integral member of the research team). Conversely, in North Macedonia, where tandem teaching is established by national primary education legislation, recruitment was primarily conducted by research team members who utilized the national network of primary schools provided by Ministry of Education and science to ensure broad national coverage. School representatives were contacted using official schools e—mail addresses, complemented with personal contacts with school personnel (e.g., generalist teachers, PE teachers, and school principals) to enhance teachers engagement in the study.

The study was conducted in strict adherence to the Declaration of Helsinki. Ethical approval for the protocol and data collection procedures was formally obtained from the Ethics Committee at the Faculty of Physical Education and Sport of Comenius University in Bratislava, Slovakia (Protocol Code 10/2024, 21 June 2024). Prior to completing the online questionnaire, all participants were provided with a comprehensive explanation of the study's purpose, their rights, and the confidentiality protocols. Informed consent was secured from every participant, who was required to explicitly agree to the outlined terms before proceeding. All collected data were anonymized to ensure participant privacy and confidentiality.

### 2.2 Data collection

The data for this study were collected using a Google Forms questionnaire distributed between June 16 and July 11, 2025. This timing was deliberately scheduled at the close of the academic year to capture reflections grounded in sustained practical experience. The questionnaire was initially developed in Macedonian, subsequently translated into English, and ultimately rendered into Slovak for deployment across both nations. The questionnaire was structured to gather data across several dimensions of tandem teaching, utilizing distinct response formats. The analysis focused on key questions, which included:

- Items for Teacher Roles (Q1 & Q2): Two multi-item sections investigated the division of labor. Question 1 assessed the teachers' level of personal involvement in various tandem teaching tasks using a categorical scale (e.g., “I am completely involved,” “We are working together with my colleague from the tandem”). Question 2 required participants to identify the person responsible for specific tasks (e.g., planning, selecting equipment), with mutually exclusive options such as “Responsibility of generalist teacher,” “Responsibility of PE specialist,” or “Mutual responsibility of both teachers in the tandem”
- Scale Questions (Q5 & Q8):
  ◦ Question 5 evaluated the overall implementation of the tandem teaching concept on a 6-point scale, ranging from 1 (Lowest level) to 6 (The highest level).
  ◦ Question 8 consisted of six multi-items where teachers rated their level of agreement with statements concerning the organization and support of tandem teaching on a 5-point scale (e.g., “Completely agree” to “Completely disagree”).
- Multi-Choice and Open-Ended Items (Q6, Q7, & Q9): Questions 6 and 7 were multi-choice, requiring participants to identify a maximum of three main benefits and challenges of tandem teaching, respectively. Finally, Question 9 was an open-ended response item, allowing teachers to express, based on their experience and opinion, specific suggestions for improving the current implementation of tandem teaching in PE.

The instrument was developed *ad hoc* for this investigation due to the absence of a standardized tool covering the unique pedagogical and structural dimensions of tandem teaching in both national contexts. To ensure the psychometric quality of the instrument, several measures for validity and reliability were implemented.

- Content and Face Validity: An internal expert committee specializing in PE and General Education oversaw the instrument's development and refinement, ensuring high content validity through comprehensive coverage of relevant topics and the prioritization of clear, unambiguous language.
- Cross-Cultural Equivalence: A formal back-translation procedure (Macedonian → English → Slovak) was executed to establish semantic and conceptual equivalence. This procedure supports the instrument's reliability and consistency across the distinct national and linguistic settings.
- Construct Validity and Reliability: The scale items used to measure the implementation factors in Question 8 were empirically tested for internal quality. The six items comprising the implementation construct demonstrated excellent internal consistency, with a Cronbach's Alpha (*α*) of 0.914. This high value indicates a strong level of inter-relatedness and reliability among the scale items. Furthermore, the data were determined to be highly suitable for factor analysis, confirmed by the Kaiser-Meyer-Olkin measure (KMO = 0.910). Principal Component Analysis (PCA) confirmed a unidimensional structure, with all six implementation statements loading strongly onto a single extracted factor, which accounted for 71.008% of the total variance (see Table 6). This evidence supports the construct validity of grouping these six statements to measure the singular construct of “*Perceived Quality of Tandem Teaching Implementation.”*

**Table 6:** Psychometric Properties of the Implementation Quality Scale: Factor Loadings, KMO, and Internal Consistency (α).

KMO and Bartlett's Test						
Kaiser-Meyer-Olkin Measure of Sampling Adequacy				.910		
Bartlett's Test of Sphericity		Approx. Chi-Square		2,537.550		
		df		15		
		Sig.		.000		
Cronbach's alpha		N of items				
.914		6				
Total variance explained						
Component	Initial eigenvalues			Extraction sums of squared loadings		
	Total	% of variance	Cumulative %	Total	% of variance	Cumulative %
1	4.260	71.008	71.008	4.260	71.008	71.008
2	.496	8.274	79.281			
3	.428	7.130	86.411			
4	.400	6.671	93.082			
5	.222	3.703	96.785			
6	.193	3.215	100.000			
Component loadings						
Variables		Communalities		Factor loading (Component 1)		
Sistematic_implementation		.597		.773		
Clear_instr_generalist		.772		.879		
Clear_instr_PEteachers		.788		.888		
Guidelines_curriculum		.670		.819		
Guidelines_expectations		.816		.903		
Support_teachers		.616		.785		

### 2.3 Data analysis

The collected empirical data were subjected to rigorous statistical analysis to address the study's comparative objectives regarding the operational structure and perceived quality of tandem teaching implementation. All statistical computations were performed using IBM SPSS Statistics software, with the threshold for statistical significance established at *α* = 0.05.

Initial data evaluation commenced with the application of descriptive statistics to characterize the sample demographics and to delineate the frequency and percentage distributions of responses across all primary instruments (Q1, Q2, Q5, Q6, Q7, and Q8).

To investigate systematic cross-national differences in the structure of the collaborative model, the relationship between the categorical variable of the country of origin (Slovakia or North Macedonia) and the response patterns for the multi-item instruments on Teacher Involvement (Q1) and Task Responsibility (Q2) was assessed. The distinct response options for both Q1 and Q2 were treated as categorical variables (ordinal for Q1 and nominal for Q2). A Chi-square test of independence was performed on every individual task item within Q1 and Q2. When a statistically significant difference in distribution was detected, Cramér's *V* was subsequently calculated to quantify the effect size and strength of the association between the country and the specific pattern of response distribution.

For instruments employing a multi-point rating scale (Q5 and Q8), distinct inferential methodologies were utilized:

Overall Implementation Evaluation (Q5): An Independent Samples *T*-test was executed to compare the mean scores of generalist teachers’ overall perception of tandem teaching implementation quality between the two countries (Slovakia and North Macedonia).

Implementation Factors Agreement (Q8): Following the validation of the construct's psychometric properties (i.e., reliability and unidimensional factor structure), an Independent Samples *T*-test was performed on the mean scores for the individual implementation statements to contrast generalist teachers' level of agreement on the organizational and supportive aspects of the program between the two national samples.

The responses for the primary benefits (Q6) and main challenges (Q7), which employed a multi-choice, closed-list format, were analyzed using descriptive statistics (frequencies and percentages) to identify the most commonly selected options by teachers in each country.

Conversely, the responses to the open-ended question concerning suggestions for improvement (Q9) were analyzed using Thematic Content Analysis (TCA) (34) and Qualitative Content Analyses (35). These methodologies were selected to facilitate the systematic categorization of emergent qualitative textual data, and to quantify the frequency of emerging thematic concepts. An inductive coding process was applied. Two researchers independently read all responses to achieve familiarisation with the data and developed an initial coding framework based on recurring ideas and suggestions. Responses were coded independently by both researchers. The coding framework was refined through discussion until consensus was reached. Codes were subsequently grouped into higher—order categories, resulting with key areas for improvement (five for North Macedonia and four for Slovakia). The analyses was conducted separately for each country to preserve contextual specificity. Answering Q9 was optional and not all participants provided open-ended responses. Therefore, findings from Q9 reflect the perspectives of teachers who chose to elaborate on their experiences and should be interpreted as illustrative rather than representative of the full sample.

## 3 Results

Given the robust dataset and the use of multiple analytical procedures, the Results section is organized into five subsections for clarity and ease of understanding. Section 3.1 presents the results of the validation of the construct's psychometric properties and examines differences between teachers from different countries in their overall perceptions of tandem teaching implementation using Independent Samples *T*-test. Sections 3.2 and 3.3. provide an overview of results for teachers` roles (3.2) and responsibilities (3.3) within the tandem, analysing frequences (f) and percentages (%) for teachers from both countries. Differences between teachers based on country level, were assessed using Chi—square test, with effect size estimated using Cramer's V test. Section 3.4. reports teachers` perceived benefits and challenges in tandem teaching, based on the top three ranked choices and analyzed using frequency counts. Finally, Section 3.5. presents teachers`suggestions for improving tandem teaching, identified through qualitative content analyses of open—ended responses.

### 3.1 Generalist teachers' overall perceptions of tandem teaching implementation

The overall perception of the tandem teaching concept was evaluated on a 6-point scale (1 = Lowest level, 6 = Highest level). An Independent Samples *T*-test revealed no statistically significant difference in the mean overall evaluation between teachers from Slovakia (*M* = 4.35, *SD* = 1.101) and North Macedonia (*M* = 4.25, *SD* = 1.019). The test statistic was *t* = 1.176 (*df* = 616, *p* = 0.240), and the mean difference was small (0.10). Levene's test confirmed the homogeneity of variances (*F* = 0.185, *p* = 0.667). These results suggest that teachers in both countries expressed similarly positive perceptions of the tandem concept's overall implementation, rating it slightly above average on the 6-point scale (see Table 7a,b).

**Table 7:** (a) Overall generalist teachers' perception of the tandem teaching concept: mean scores and standard deviations. (b) Independent samples *T*-test for generalist teachers’ overall perception of the tandem teaching concept by country (Slovakia vs. North Macedonia)

Evaluation of the overall implementation of the concept of tandem teaching in PE		Country	*N*		Mean		Std. Deviation	Std. Error Mean	
	Slovakia			314	4,35		1,101	,062	
	North Macedonia			304	4,25		1,019	,058	
Independent samples test									
Evaluation of the overall implementation of the concept of tandem teaching in PE	Levene's test for equality of variances		*T*-test for equality of means						
	F	Sig.	t	df	Sig. (2-tailed)	Mean Difference	Std. Error Difference	95% Confidence Interval of the Difference	
								Lower	Upper
Equal variances assumed	.185	.667	1.176	616	.240	.100	.085	(.067)	.268
Equal variances not assumed			1.178	614.768	.239	.100	.085	(.067)	.268

The underlying structure of the scale items demonstrated excellent psychometric properties, confirming a unidimensional construct for implementation quality. This was validated by a PCA. Sampling adequacy was confirmed by a KMO value of 0.910 and a significant Bartlett's Test of Sphericity [*χ*^2^(15) = 2537.55, *p* < 0.001], confirming data suitability for factor analysis. The PCA extracted one strong component with an eigenvalue greater than 1, explaining 71.01% of the total variance. All six items loaded highly (ranging from 0.773 to 0.903) on this single component, confirming a unidimensional structure. The internal consistency of the scale was excellent, with a Cronbach's α coefficient of 0.914, thereby establishing the reliability of the construct (Table 6).

An Independent Samples *T*-test was then conducted on the individual factor items to examine potential cross-national differences in teachers' level of agreement regarding the quality of the implementation process (Table 8). Statistically significant differences were found on three key factors, with teachers from North Macedonia reporting consistently higher agreement levels than their Slovakian counterparts:

- Systematic implementation: *p* < 0.001 (Mean Diff = 0.288)
- Support for teachers: *p* = 0.001 (Mean Diff = 0.360)
- Guidelines for curriculum implementation: *p* = 0.012 (Mean Diff = 0.223)

**Table 8:** Cross-National Differences in Perceived Implementation Quality: Independent Samples *T*-Test on Factor Items by Country.

Variables	Mean diff	*p*-value
Sistematic_implementation	0.288	0.000
Clear_instr_generalist	0.063	0.509
Clear_instr_PEteachers	0.095	0.279
Guidelines_curriculum	0.223	0.012
Guidelines_expectations	0.082	0.357
Support_teachers	0.360	0.001

*p* < .05.

The findings reveal that teachers across both contexts generally perceived the tandem teaching implementation process positively, particularly in terms of clarity of guidelines and collaboration between generalist and PE teachers. The psychometric results (PCA and Cronbach's *α*) indicate that these perceptions form a coherent and unified construct, suggesting that systematic planning, clear guidance, and support are highly interrelated components of perceived successful implementation.

### 3.2 Generalist teachers' task involvement

Tandem teaching in PE introduces new roles and responsibilities for the teachers involved. Given the novelty of the concept and the modified roles, one of the aims of our study was to identify the level of personal involvement (Q1) and compare the two tandem models in North Macedonia (generalist teacher/PE specialist) and Slovakia (generalist teacher/coach).

Teacher involvement was investigated across 15 tasks organized into five main areas: instructional planning and content design, logistics and environment, classroom and behavior management, instructional delivery and assessment, and student support and engagement. Participants reported their level of personal involvement using a categorical scale (e.g., “I am not involved at all,” “I am completely involved,” or “We are working together with my colleague from the tandem”). The full list of 15 tasks is presented in Table 9, as well as the distribution of responses.

**Table 9:** Generalist teachers' self-reported involvement in tandem tasks (Q1): frequencies and percentages by task area and country.

Variable	Category	Slovakia	North Macedonia	Overall
		*n* (%) 314 (50.8%)	*n* (%) 304 (49.2%)	*n* (%) 618 (100%)
1. Planning of the content of PE lessons	I am not involved at all	99 (31.5%)	18 (5.9%)	117 (18.9%)
	I am partly involved	86 (27.4%)	47 (15.5%)	133 (21.5%)
	I am completely involved	33 (10.5%)	120 (39.5%)	153 (24.8%)
	We are working together with my colleague from the tandem	27 (8.6%)	104 (34.2%)	131 (21.2%)
	We are working by mutual understanding based on available time	69 (22%)	15 (4.9%)	84 (13.6%)
2. Choosing activities and content that should be implemented, including exercises and games	I am not involved at all	96 (30.6%)	18 (5.9%)	114 (18.4%)
	I am partly involved	91 (29.0%)	53 (17.4%)	144 (23.3%)
	I am completely involved	52 (16.6%)	118 (38.8%)	170 (27.5%)
	We are working together with my colleague from the tandem	21 (6.7%)	99 (32.6%)	120 (19.4%)
	We are working by mutual understanding based on available time	54 (17.2%)	16 (5.3%)	70 (11.3%)
3. Decision for the location where the PE lesson should be delivered	I am not involved at all	70 (22.3%)	20 (6.6%)	90 (14.6%)
	I am partly involved	55 (17.5%)	40 (13.2%)	95 (15.4%)
	I am completely involved	90 (28.7%)	114 (37.5%)	204 (33.0%)
	We are working together with my colleague from the tandem	33 (10.5%)	103 (33.9%)	136 (22.0%)
	We are working by mutual understanding based on available time	66 (21.0%)	27 (8.9%)	93 (15.0%)
4. Selection of the equipment needed for PE lesson	I am not involved at all	104 (33.1%)	31 (10.2%)	135 (21.8%)
	I am partly involved	85 (27.1%)	44 (14.5%)	129 (20.9%)
	I am completely involved	47 (15.0%)	122 (40.1%)	169 (27.3%)
	We are working together with my colleague from the tandem	27 (8.6%)	92 (30.3%)	119 (19.3%)
	We are working by mutual understanding based on available time	51 (16.2%)	15 (4.9%)	66 (10.7%)
5. Selection of organizational forms for PE lessons	I am not involved at all	102 (32.5%)	28 (9.2%)	130 (21.0%)
	I am partly involved	87 (27.7%)	41 (13.5%)	128 (20.7%)
	I am completely involved	46 (14.6%)	121 (39.8%)	167 (27.0%)
	We are working together with my colleague from the tandem	31 (9.9%)	105 (34.5%)	136 (22.0%)
	We are working by mutual understanding based on available time	48 (15.3%)	9 (3.0%)	57 (9.2%)
6. Preparing students for PE lesson	I am not involved at all	31 (9.9%)	19 (6.3%)	50 (8.1%)
	I am partly involved	60 (19.1%)	47 (15.5%)	107 (17.3%)
	I am completely involved	127 (40.4%)	127 (41.8%)	254 (41.1%)
	We are working together with my colleague from the tandem	43 (13.7%)	96 (31.6%)	139 (22.5%)
	We are working by mutual understanding based on available time	53 (16.9%)	15 (4.9%)	68 (11.0%)
7. Keeping order and discipline during PE class	I am not involved at all	9 (2.9%)	8 (2.6%)	17 (2.8%)
	I am partly involved	36 (11.5%)	41 (13.5%)	77 (12.5%)
	I am completely involved	110 (35.0%)	132 (43.4%)	242 (39.2%)
	We are working together with my colleague from the tandem	100 (31.8%)	110 (36.2%)	210 (34.0%)
	We are working by mutual understanding based on available time	59 (18.8%)	13 (4.3%)	72 (11.7%)
8. Practical demonstration of PE contents and activities	I am not involved at all	45 (14.3%)	21 (6.9%)	66 (10.7%)
	I am partly involved	91 (29.0%)	70 (23.0%)	161 (26.1%)
	I am completely involved	67 (21.3%)	122 (40.1%)	189 (30.6%)
	We are working together with my colleague from the tandem	60 (19.1%)	71 (23.4%)	131 (21.2%)
	We are working by mutual understanding based on available time	51 (16.2%)	20 (6.6%)	71 (11.5%)
9. Explanation of tasks, activities, roles and rules during movement and sport games	I am not involved at all	43 (13.7%)	21 (6.9%)	64 (10.4%)
	I am partly involved	88 (28.0%)	72 (23.7%)	160 (25.9%)
	I am completely involved	66 (21%)	123 (40.5%)	189 (30.6%)
	We are working together with my colleague from the tandem	69 (22%)	68 (22.4%)	137 (22.2%)
	We are working by mutual understanding based on available time	48 (15.3%)	20 (6.6%)	68 (11.0%)
10. Identifying mistakes in children`s performance and correction	I am not involved at all	16 (5.1%)	18 (5.9%)	34 (5.5%)
	I am partly involved	75 (23.9%)	59 (19.4%)	134 (21.7%)
	I am completely involved	88 (28.0%)	135 (44.4%)	223 (36.1%)
	We are working together with my colleague from the tandem	81 (25.8%)	78 (25.7%)	159 (25.7%)
	We are working by mutual understanding based on available time	54 (17.2%)	14 (4.6%)	68 (11.0%)
11. Work with children with difficulties or special needs	I am not involved at all	23 (7,3%)	16 (5.3%)	39 (6.3%)
	I am partly involved	41 (13,1%)	56 (18.4%)	97 (15.7%)
	I am completely involved	98 (31,2%)	108 (35.5%)	206 (33.3%)
	We are working together with my colleague from the tandem	89 (28,3%)	114 (37.5%)	203 (32.8%)
	We are working by mutual understanding based on available time	63 (20.1%)	10 (3.3%)	73 (11.8%)
12. Motivation, encouragement and emotional support for children	I am not involved at all	14 (4.5%)	14 (4.6%)	28 (4.5%)
	I am partly involved	46 (14.6%)	39 (12.8%)	85 (13.8%)
	I am completely involved	97 (30.9%)	127 (41.8%)	224 (36.2%)
	We are working together with my colleague from the tandem	93 (29.6%)	116 (38.2%)	209 (33.8%)
	We are working by mutual understanding based on available time	64 (20.4%)	8 (2.6%)	72 (11.7%)
13. Maintaining the socio-emotional climate in the classroom during the PE lessons	I am not involved at all	11 (3.5%)	11 (3.6%)	22 (3.6%)
	I am partly involved	39 (12.4%)	46 (15.1%)	85 (13.8%)
	I am completely involved	104 (33.1%)	117 (38.5%)	221 (35.8%)
	We are working together with my colleague from the tandem	99 (31.5%)	120 (39.5%)	219 (35.4%)
	We are working by mutual understanding based on available time	61 (19.4%)	10 (3.3%)	71 (11.5%)
14. Communication with parents for issues related to children's physical health and behavior during PE lessons and extracurricular activities related to PE	I am not involved at all	28 (8.9%)	15 (4.9%)	43 (7.0%)
	I am partly involved	44 (14.0%)	59 (19.4%)	103 (16.7%)
	I am completely involved	159 (50.6%)	104 (34.2%)	263 (42.6%)
	We are working together with my colleague from the tandem	41 (13.1%)	111 (36.5%)	152 (24.6%)
	We are working by mutual understanding based on available time	42 (13.4%)	15 (4.9%)	57 (9.2%)
15. Implementing activities that provide holistic learning and correlation links between PE and other subjects	I am not involved at all	35 (11.1%)	21 (6.9%)	56 (9.1%)
	I am partly involved	68 (21.7%)	46 (15.2%)	114 (18.5%)
	I am completely involved	103 (32.8%)	103 (34.0%)	206 (33.4%)
	We are working together with my colleague from the tandem	54 (17.2%)	115 (38.0%)	169 (27.4%)
	We are working by mutual understanding based on available time	54 (17.2%)	18 (5.9%)	72 (11.7%)

In relation to instructional planning and content design, overall, slight differences are notable between countries. In the cohort of participants from North Macedonia, the greater proportion of teachers indicated “being completely involved“ in planning (39.5%), choosing activities and contents for the lessons (38.8%), selection of organizational forms (39.8%) and implementing activities for holistic learning (38%), followed by “working together with my colleagues from the tandem” on planning (34.2%), selection of activities (32.6%); selection of organizational forms (34.5%); holistic learning (38%). In contrary, in the cohort of Slovak teachers, the greater proportion of teachers indicated being “not involved at all” in planning (31.5%), choosing activities and contents for the lessons (30.6%), selection of organizational forms (32.5%), followed by second larger proportion of respondents claiming being “partly involved” in planning (27.4%), selection of organizational forms (27.7) and selection of activities (29.9%). Slight differences in Slovak teachers are noted for involvement in activities that provide holistic learning, where 32.8% are “completely involved”, 21% partly involved, and 17% are “working together with a tandem colleague” or “work based on mutual understanding”. These findings suggest that Macedonian teachers perceive planning and content design as a shared responsibility, while for Slovak teachers, it is more an individual responsibility of one of the teachers, pointing to potentially inconsistent collaboration practices.

In terms of logistic and environment covering decisions related to the physical setup and resources for PE lessons, the greater proportion of teachers in both groups reported being “completely involved” in the decision for the location of the PE lesson (28% Slovak, 37.5% Macedonian teachers) and preparation of students for PE lessons (40.4% Slovak, 41.8% Macedonian teachers). The second largest choice indicates slight differences between teachers from both countries in a sense of “not being involved” in a case of Slovak teachers (22.3% when choosing location, 19% in preparing students), compared to their Macedonian colleagues that are “working together with tandem teachers” when selecting the location (33.9%) and preparation of students (31.6%). Similarly, teachers from North Macedonia (40.1%) are “completely involved” or “work together with the colleague from the tandem” (30.3%) when selecting the equipment for PE lessons, while the majority of Slovak teachers “are not involved at all” (33.1%) or “partly involved” (27.1%) in this task. Presented findings indicate that while both groups recognize the importance of teacher involvement in lesson logistics, Slovak teachers tend to perceive these responsibilities as delegated or limited, whereas Macedonian teachers highlight shared responsibility and cooperative practice. This points to differences in how tandem teaching is operationalized across the two contexts: more individualized or specialist-driven in Slovakia, and more collaborative in North Macedonia.

Across the items related to classroom and behavior management, the highest proportion of respondents from both countries reported being “completely involved” keeping order and discipline during PE classes (35% of Slovak teachers, 43.4% of teachers from North Macedonia), followed up with 31.8% of teachers in Slovakia and 36% of their Macedonian colleagues that work on this “together with the colleagues from the tandem”. On the other two items related to this category, maintaining the socio—emotional climate during PE lessons and communication with parents, the greatest proportion of teachers from N. Macedonia are “working together with the colleagues from the tandem” (39.5% for maintaining socio—emotional climate and 36.5% for communication with parents), followed by 38.5% and 34.2% of respondents referring to be “completely involved” in above mentioned tasks. In the case of Slovak teachers, the greater proportion of respondents reported being “completely involved” in maintaining class climate (33.1%) and communicating with parents (50.6%). Similar to findings of previous sections, the results underline the strong engagement in classroom and behavior management during PE lessons, underlining the emphasis of teachers from North Macedonia toward collaborative approaches within the tandem model and a more individualized perception of responsibility in these areas by Slovak teachers.

The aspect of instructional delivery and assessment represented through practical demonstration of PE contents and activities, explanation of tasks, roles, and rules during movement and games, identifying mistakes and providing correction, and working with children with difficulties or special needs also supports slight differences between teachers from both countries. In this regard, the majority of Slovak teachers reported being “partly involved” in practical demonstration (29%), explanation of games and tasks (28%), followed by a smaller number of being “completely involved” in practical demonstration (21.3%) and explanation of games and roles (21%). In the cohort of Macedonian teachers, the highest proportion reported being “completely involved” in practical demonstration of PE contents (40.1%), explanation of tasks and games (40.5%), and identifying mistakes in children's performance (44.4%). These responses are followed by a nearly equal proportion of statements for being “partly involved” (23% and 23.7% respectively, for demonstration and explanation of roles) and “working together with the colleague from the tandem” (23.4% and 22.4%, respectively). When it comes to identifying mistakes in performance, the second highest proportion of teachers from Macedonia (25.7%) and Slovakia (25.8%) “work together with colleagues from the tandem”. Regarding the work with children with special educational needs, teachers from both countries are “completely involved” individually and in cooperation, “working with the colleague from the tandem” (Table 9). These results indicate that Macedonian teachers perceive themselves as more directly responsible for instructional delivery, while Slovak teachers more often report partial involvement, reflecting a tendency toward shared or delegated responsibility. At the same time, both groups demonstrate strong engagement in supporting children with special needs, underscoring the importance of inclusivity as a common priority across contexts.

Overall, the presented findings suggest a consistent trend of slightly different approaches toward tasks within the tandem, recognizing the tendency toward shared responsibility and cooperative practice among teachers from North Macedonia and a more individualized approach and perception of responsibilities among Slovak teachers.

The differences in perceptions of tasks in PE tandem teaching between teachers in Slovakia and North Macedonia were identified using Chi—square test, while the effect size was estimated using Cramer's *V* test. These results are presented in Table 10. The analyses of the Chi-square test revealed statistically significant differences between teachers from Slovakia and North Macedonia across all examined aspects of PE tandem teaching (*p* < .001). However, the strength of association, measured by Cramér's *V*, varied across variables, indicating that some areas showed stronger divergence between the two groups than others. Strong associations were identified for aspects related to planning of the content of the PE lesson (*V* = .564), choosing activities and content to be implemented (*V* = .509), and selection of organizational forms for PE lessons (*V* = .507). These results suggest that teachers from the two countries differed considerably in how they perceived their tasks in structuring and organizing PE lessons within the tandem teaching framework. Moderate associations were observed for the aspect of logistics and environment and communication with parents. Moderate association is noted in decisions regarding location for delivery of PE lessons (*V* = .371), selection of appropriate equipment (*V* = .477), and communication with parents (*V* = .318). Weaker associations were found in other aspects related to classroom behavior and management, such as keeping order and discipline (*V* = .228) and maintaining socio–emotional climate during PE lessons (*V* = .254) and aspects related to instructional delivery of PE lessons. These include practical demonstrations (*V* = .260), explaining tasks and rules (*V* = .247), identifying mistakes in children's performance (*V* = .239), working with children with special needs (*V* = .271). These weaker associations suggest that teachers from both countries held relatively similar perceptions regarding their hands-on responsibilities during lesson delivery and classroom behavior. Overall, the results indicate that national differences were most evident in planning and organizational domains, while classroom management and interactional responsibilities were perceived more uniformly.

**Table 10:** Inferential analysis of task involvement (Q1): Chi-square and cramér's V test results.

Variable	Pearson chi-square (*N*)	df	*p*-value	Cramér's V^a^	Association strength
1. Planning of the content of PE lesson	196,85 (618)	4	.000	.564	Strong
2. Choosing activities and content that should be implemented, including exercises and games	160,228 (618)	4	.000	.509	Strong
3. Decision for the location where the PE lesson should be delivered	85,214 (618)	4	.000	.371	Medium
4. Selection of the equipment needed for PE lesson	140,805 (618)	4	.000	.477	Medium
5. Selection of organizational forms for PE lessons	159,166 (618)	4	.000	.507	Strong
6. Preparing students for PE lesson	45,754 (618)	4	.000	.272	Weak
7. Keeping order and discipline during PE class	32,095 (618)	4	.000	.228	Weak
8 Practical demonstration of PE contents and activities	41,780 (618)	4	.000	.260	Weak
9. Explanation of tasks, activities, roles and rules during movement and sport games	37,738 (618)	4	.000	.247	Weak
10. Identifying mistakes in children`s performance and correction	35,367 (618)	4	.000	.239	Weak
11. Work with children with difficulties or special needs	45,470 (618)	4	.000	.271	Weak
12. Motivation, encouragement and emotional support for children	50,532 (618)	4	.000	.286	Weak
13. Maintaining the socio-emotional climate in the classroom during the PE lessons	39,837 (618)	4	.000	.254	Weak
14. Communication with parents for issues related to children's physical health and behavior during PE lessons and extracurricular activities related to PE	62,497 (618)	4	.000	.318	Medium
15. Implementing activities that provide holistic learning and correlation links between PE and other subjects	47,582 (618)	4	.000	.278	Weak

a Weak (small) effect 0.10–0.29; Moderate (medium) 0.30–0.49; Strong (large effect) ≥0.50.

### 3.3 Generalist teachers' responsibilities

Another aspect of interest of our study was the perception of responsibilities of teachers within tandem teaching and in this regard, the differences between the tandem models in Slovakia and North Macedonia. Teachers shared their responsibilities within 15 aspects related to instructional planning and content design, logistics and environment, classroom and behavior management, instructional delivery and assessment and student support and engagement. Teacher Responsibilities (Q2) were reported using options such as “Responsibility of generalist teacher,” “Responsibility of PE specialist/coach”, “Mutual responsibility of both teachers in the tandem”, “Depending on the situation and available time”, “It`s not clearly defined,” and “It`s a mutual understanding and agreement”. The full list of responsibilities and distribution of responses is presented in Table 11.

**Table 11:** Generalist teachers' perception of task responsibility (Q2): frequencies and percentages of responsibility allocation by task area and country.

Variable	Category	Slovakia	North Macedonia	Overall
		*n* (%) 314 (50,8%)	*n* (%) 304 (49,2%)	*n* (%) 618 (100%)
1. Planning of the content of PE lessons	Responsibility of generalist teacher	22 (7%)	17 (5,6%)	36 (6,3%)
	Responsibility of PE specialist/ coach	153 (48,7%)	48 (15,8%)	201 (32,5%)
	Mutual responsibility of both teachers in the tandem	84 (26,8%)	228 (75,0%)	312 (50,5%)
	Depending from the situation and available time	21 (6,7%)	3 (1.0%)	24 (3,9%)
	It`s not clearly defined	17 (5,4%)	3 (1,0%)	20 (3,2%)
	It`s a mutual understanding and agreement	17 (5,4%)	5 (1,6%)	22 (3,6%)
2. Choosing activities and content that should be implemented, including exercises and games	Responsibility of generalist teacher	4 (1,3%)	8 (2,6%)	12 (1,9%)
	Responsibility of PE specialist/ coach	192 (61,1%)	106 (34,9%)	298 (48,2%)
	Mutual responsibility of both teachers in the tandem	72 (22,9%)	171 (56,3%)	243 (39,3%)
	Depending from the situation and available time	15 (4,8%)	9 (3,0%)	24 (3,9%)
	It`s not clearly defined	7 (2.2%)	2 (0,7%)	9 (1,5%)
	It`s a mutual understanding and agreement	24 (13,1%)	8 (2,6%)	32 (5,2%)
3. Decision for the location where the PE lesson should be delivered	Responsibility of generalist teacher	41 (13,1%)	6 (2,0%)	47 (7,6%)
	Responsibility of PE specialist/ coach	83 (26,5%)	103 (33,9%)	186 (30,1%)
	Mutual responsibility of both teachers in the tandem	120 (38,2%)	177 (58,2%)	297 (48,1%)
	Depending from the situation and available time	37 (11,8%)	9 (3,0%)	46 (7,4%)
	It`s not clearly defined	7 (2,2%)	0 (0,0%)	7 (1,1%)
	It`s a mutual understanding and agreement	26 (8,3%)	9 (3,0%)	35 (5,7%)
4. Selection of the equipment needed for PE lesson	Responsibility of generalist teacher	2 (0,6%)	5 (1,6%)	7 (1,1%)
	Responsibility of PE specialist/ coach	202 (64,3%)	138 (45,4%)	340 (55,0%)
	Mutual responsibility of both teachers in the tandem	73 (23,2%)	144 (47,4%)	217 (35,1%)
	Depending from the situation and available time	11 (3,5%)	9 (3,0%)	20 (3,2%)
	It`s not clearly defined	4 (1,3%)	0 (0,0%)	4 (0,6%)
	It`s a mutual understanding and agreement	22 (7,0%)	8 (2,6%)	30 (4,9%)
5. Selection of organizational forms for PE lessons	Responsibility of generalist teacher	4 (1,3%)	3 (1,0%)	7 (1,1%)
	Responsibility of PE specialist/ coach	181 (57,6%)	127 (41,8%)	308 (49,8%)
	Mutual responsibility of both teachers in the tandem	91 (29%)	166 (54,6%)	257 (41,6%)
	Depending from the situation and available time	12 (3,8%)	2 (0,7%)	14 (2,3%)
	It`s not clearly defined	5 (1,6%)	0 (0,0%)	5 (0,8%)
	It`s a mutual understanding and agreement	21 (6,7%)	6 (2,0%)	27 (4,4%)
6. Preparing students for PE lesson	Responsibility of generalist teacher	136 (22,0%)	48 (7,8%)	184 (29,8%)
	Responsibility of PE specialist/ coach	44 (7,1%)	29 (4,7%)	73 (11,8%)
	Mutual responsibility of both teachers in the tandem	102 (16,5%)	221 (35,8%)	323 (52,3%)
	Depending from the situation and available time	10 (1,6%)	2 (0,3%)	12 (1,9%)
	It`s not clearly defined	6 (1,0%)	0 (0,0%)	6 (1,0%)
	It`s a mutual understanding and agreement	16 (2,6%)	4 (0,6%)	20 (3,2%)
7. Keeping order and discipline during PE class	Responsibility of generalist teacher	33 (5,3%)	22 (3,6%)	55 (8,9%)
	Responsibility of PE specialist/ coach	20 (3,2%)	44 (7,1%)	64 (10,4%)
	Mutual responsibility of both teachers in the tandem	231 (37,4%)	229 (37,1%)	460 (74,4%)
	Depending from the situation and available time	5 (0,8%)	4 (0,6%)	9 (1,5%)
	It`s not clearly defined	5 (0,8%)	0 (0,0%)	5 (0,8%)
	It`s a mutual understanding and agreement	20 (3,2%)	5 (0,8%)	25 (4,0%)
8. Practical demonstration of PE contents and activities	Responsibility of generalist teacher	7 (1,1%)	7 (1,1%)	14 (2,3%)
	Responsibility of PE specialist/ coach	138 (22,3%)	153 (24,8%)	291 (47,1%)
	Mutual responsibility of both teachers in the tandem	139 (22,5%)	128 (20,7%)	267 (43,2%)
	Depending from the situation and available time	9 (1,5%)	8 (1,3%)	17 (2,8%)
	It`s not clearly defined	4 (0,6%)	0 (0,0%)	4 (0,6%)
	It`s a mutual understanding and agreement	17 (2,8%)	8 (1,3%)	25 (4,0%)
9. Explanation of tasks, activities, roles and rules during movement and sport games	Responsibility of generalist teacher	9 (1,5%)	9 (1,5%)	18 (2,9%)
	Responsibility of PE specialist/ coach	136 (22,0%)	142 (23,0%)	278 (45,0%)
	Mutual responsibility of both teachers in the tandem	145 (23,5%)	136 (22,0%)	281 (45,5%)
	Depending from the situation and available time	5 (0,8%)	7 (1,1%)	12 (1,9%)
	It`s not clearly defined	3 (0,5%)	0 (0,0%)	3 (0,5%)
	It`s a mutual understanding and agreement	16 (2,6%)	10 (1,6%)	26 (4,2%)
10. Identifying mistakes in children`s performance and correction	Responsibility of generalist teacher	12 (1,9%)	8 (1,3%)	20 (3,2%)
	Responsibility of PE specialist/ coach	44 (7,1%)	109 (17,6%)	153 (24,8%)
	Mutual responsibility of both teachers in the tandem	222 (35,9%)	169 (27,3%)	391 (63,3%)
	Depending from the situation and available time	7 (1,1%)	10 (1,6%)	17 (2,8%)
	It`s not clearly defined	6 (1.0%)	0 (0.0%)	6 (1.0%)
	It`s a mutual understanding and agreement	23 (3.7%)	8 (1.3%)	31 (5.0%)
11. Work with children with difficulties or special needs	Responsibility of generalist teacher	42 (6.8%)	17 (2.8%)	59 (9.5%)
	Responsibility of PE specialist/ coach	23 (3.7%)	44 (7.1%)	67 (10.8%)
	Mutual responsibility of both teachers in the tandem	201 (32.5%)	224 (36.2%)	425 (68.8%)
	Depending from the situation and available time	14 (2.3%)	2 (0.3%)	16 (2.6%)
	It`s not clearly defined	8 (1.3%)	7 (1.1%)	15 (2.4%)
	It`s a mutual understanding and agreement	26 (4.2%)	10 (1.6%)	36 (5.8%)
12. Motivation, encouragement and emotional support for children	Responsibility of generalist teacher	10 (1.6%)	10 (1.6%)	20 (3.2%)
	Responsibility of PE specialist/ coach	36 (5.8%)	68 (11.0%)	104 (16.8%)
	Mutual responsibility of both teachers in the tandem	233 (37.7%)	217 (35.1%)	450 (72.8%)
	Depending from the situation and available time	9 (1.5%)	4 (0.6%)	13 (2.1%)
	It`s not clearly defined	4 (0.6%)	0 (0.0%)	4 (0.6%)
	It`s a mutual understanding and agreement	22 (3.6%)	5 (0.8%)	27 (4.4%)
13. Maintaining the socio-emotional climate in the classroom during the PE lessons	Responsibility of generalist teacher	20 (3.2%)	23 (3.7%)	43 (7.0%)
	Responsibility of PE specialist/ coach	26 (4.2%)	52 (8.4%)	78 (12.6%)
	Mutual responsibility of both teachers in the tandem	233 (37.7%)	220 (35.6%)	453 (73.3%)
	Depending from the situation and available time	9 (1.5%)	4 (0.6%)	13 (2.1%)
	It`s not clearly defined	5 (0.8%)	0 (0.0%)	5 (0.8%)
	It`s a mutual understanding and agreement	21 (3.4%)	5 (0.8%)	26 (4.2%)
14. Communication with parents for issues related to children's physical health and behavior during PE lessons and extracurricular activities related to PE	Responsibility of generalist teacher	171 (27.7%)	40 (6.5%)	211 (34.1%)
	Responsibility of PE specialist/ coach	12 (1.9%)	39 (6.3%)	51 (8.3%)
	Mutual responsibility of both teachers in the tandem	82 (13.3%)	214 (34.6%)	296 (47.9%)
	Depending from the situation and available time	15 (2.4%)	2 (0.3%)	17 (2.8%)
	It`s not clearly defined	16 (2.6%)	2 (0.3%)	18 (2.9%)
	It`s a mutual understanding and agreement	18 (2.9%)	7 (1.1%)	25 (4.0%)
15. Implementing activities that provide holistic learning and correlation links between PE and other subjects	Responsibility of generalist teacher	67 (10.8%)	30 (4.9%)	97 (15.7%)
	Responsibility of PE specialist/ coach	34 (5.5%)	53 (8.6%)	87 (14.1%)
	Mutual responsibility of both teachers in the tandem	155 (25.1%)	211 (34.1%)	366 (59.2%)
	Depending from the situation and available time	17 (2.8%)	5 (0.8%)	22 (3.6%)
	It`s not clearly defined	24 (3.9%)	0 (0.0%)	24 (3.9%)
	It`s a mutual understanding and agreement	17 (2.8%)	5 (0.8%)	22 (3.6%)

Chi–square analyses were conducted to examine the differences between teachers from Slovakia and North Macedonia related to their perceptions of responsibilities in PE tandem teaching. The size of the effect was estimated using Cramer's *V* test. The effect size was determined as follows: weak (small) effect 0.10–0.29; moderate (medium) 0.30–0.49; strong (large effect) ≥0.50. These results are presented in Table 12. They revealed statistically significant differences at the level (*p* < .001) in most of the examined aspects, with the strength of association (Cramér's *V*) ranging from weak to large (.090 to.540), indicating that the magnitude of difference varied depending on the specific domain of teaching responsibility.

**Table 12:** Inferential analysis of task responsibility (Q2): Chi-square and cramér's V test results.

Variable	Pearson chi-square (*N*)	df	p- value	Cramér's V^a^	Association strength
1. Planning of the content of PE lesson	151,677	5	.000	.495	Medium
2. Choosing activities and content that should be implemented, including exercises and games	78,622	5	.000	.357	Medium
3. Decision for the location where the PE lesson should be delivered	71,311	5	.000	.340	Medium
4. Selection of the equipment needed for PE lesson	47,147	5	.000	.276	Weak
5. Selection of organizational forms for PE lessons	51,825	5	.000	.290	Weak
6. Preparing students for PE lesson	107,411	5	.000	.417	Medium
7. Keeping order and discipline during PE class	25,165	5	.000	.202	Weak
8 Practical demonstration of PE contents and activities	8,366	5	.137	.116	Weak
9. Explanation of tasks, activities, roles and rules during movement and sport games	4,975	5	.419	.090	Weak
10. Identifying mistakes in children`s performance and correction	49,237	5	.000	.282	Weak
11. Work with children with difficulties or special needs	34,445	5	.000	.236	Weak
12. Motivation, encouragement and emotional support for children	26,887	5	.000	.209	Weak
13 Maintaining the socio-emotional climate in the classroom during the PE lessons	25,863	5	.000	.205	Weak
14. Communication with parents for issues related to children's physical health and behavior during PE lessons and extracurricular activities related to PE	180,046	5	.000	.540	Large
15. Implementing activities that provide holistic learning and correlation links between PE and other subjects	63,777	5	.000	.321	Medium

a Weak (small) effect 0.10–0.29; Moderate (medium) 0.30–0.49; Strong (large effect) ≥0.50.

Statistically significant but weak association between country and teachers’ perceptions were observed in several aspects of PE tandem teaching, including selection of equipment (*χ*^2^ = 47.15, *df* = 5, *p* < .001, Cramér's *V* = .276), selection of organizational forms (*χ*^2^ = 51.83, *df* = 5, *p* < .001, Cramér's *V* = .290), maintaining discipline (*χ*^2^ = 25,16, *df* = 5, *p* < .001, Cramér's *V* = .202), identifying and correcting mistakes (*χ*^2^ = 49,24, *df* = 5, *p* < .001, Cramér's *V* = .282), working with children with special needs (*χ*^2^ = 34,45, *df* = 5, *p* < .001, Cramér's *V* = .236), motivating and emotionally supporting students (*χ*^2^ = 26,89, *df* = 5, *p* < .001, Cramér's *V* = .209), and maintaining the socio-emotional classroom climate (*χ*^2^ = 25,86, *df* = 5, *p* < .001, Cramér's *V* = .205). This result suggests that although both groups may face similar practical considerations regarding available equipment, selection of organizational forms, there could still be slight differences in who takes responsibility for organizing and preparing materials within tandem teams. Both group of teachers also perceive their responsibility for maintaining discipline in relatively similar ways. In terms of identifying mistakes in children's performance and providing correction and working with children with difficulties or special needs, the weak but significant associations suggest that core pedagogical principles of individual support and corrective feedback are shared professional norms in both contexts, though minor differences may reflect varying levels of training or resources for inclusive and adaptive teaching.

The weakest relationships were found for practical demonstration of PE content (*χ*^2^ = 8.37, *df* = 5, *p* = .137, Cramér's *V* = .116) and explanation of tasks and rules (*χ*^2^ = 4.98, *df* = 5, *p* = .419, Cramér's *V* = .090), where differences between countries were not statistically significant. These findings suggest that teachers in Slovakia and North Macedonia perceive their immediate, hands-on teaching responsibilities in relatively similar ways.

According to Cramér's *V* values, statistically significant differences (*p* < .001) and a medium association between country and teachers’ perceptions between Slovak and Macedonian teachers were identified in: planning of PE lessons, choosing activities and contents, decision for location for PE classes, preparing students and implementing activities for holistic learning. For planning the content of the PE lesson (*χ*^2^ = 151.68, *df* = 5, *p* < .001, Cramér's *V* = .495), the results suggest a stronger medium association, pointing to substantial variation between the two groups in how they perceive and divide planning responsibilities. The variables choosing activities and content for implementation (*χ*^2^ = 78.62, *df* = 5, *p* < .001, Cramér's *V* = .357) and deciding on the location of the PE lesson (*χ*^2^ = 71.31, *df* = 5, *p* < .001, Cramér's *V* = .340) also demonstrated medium associations, suggesting that teachers from the two countries differ moderately in these organizational decisions. A moderate association for preparing students for the PE lesson (*χ*^2^ = 107.41, *df* = 5, *p* < .001, Cramér's *V* = .417) indicates differences in how teachers engage students before the start of lessons. This could include variations in warm-up organization, communication of lesson goals, etc. Implementing activities that promote holistic learning and cross-curricular connections (*χ*^2^ = 63.78, *df* = 5, *p* < .001, Cramér's *V* = .321) suggests that Slovak and Macedonian teachers differ in their perceived responsibility for integrating PE with other subjects.

A large and significant association was found for communication with parents about children's physical health and behavior (*χ*^2^ = 180.05, *df* = 5, *p* < .001, Cramér's *V* = .540). This finding indicates substantial differences between Slovak and Macedonian teachers regarding this responsibility.

In summary, differences between Slovakian and Macedonian tandem teachers were most evident in preparatory responsibilities, moderately visible in organizational tasks, and minimal or absent in instructional and classroom management behaviors. The largest differences between Slovak and Macedonian tandem teachers are rooted in the strategic and administrative domains (planning, communication, coordination), while more direct instructional and classroom management tasks are perceived similarly.

### 3.4 Generalist teachers' perceptions of tandem teaching benefits and challenges

Teachers involved in tandem teaching are in a position to offer first-hand insights into the benefits of the concept. A list of nine benefits and eight perceived challenges was provided, from which teachers were asked to select the three most relevant ones based on their practical experience.

**Figure 1:**
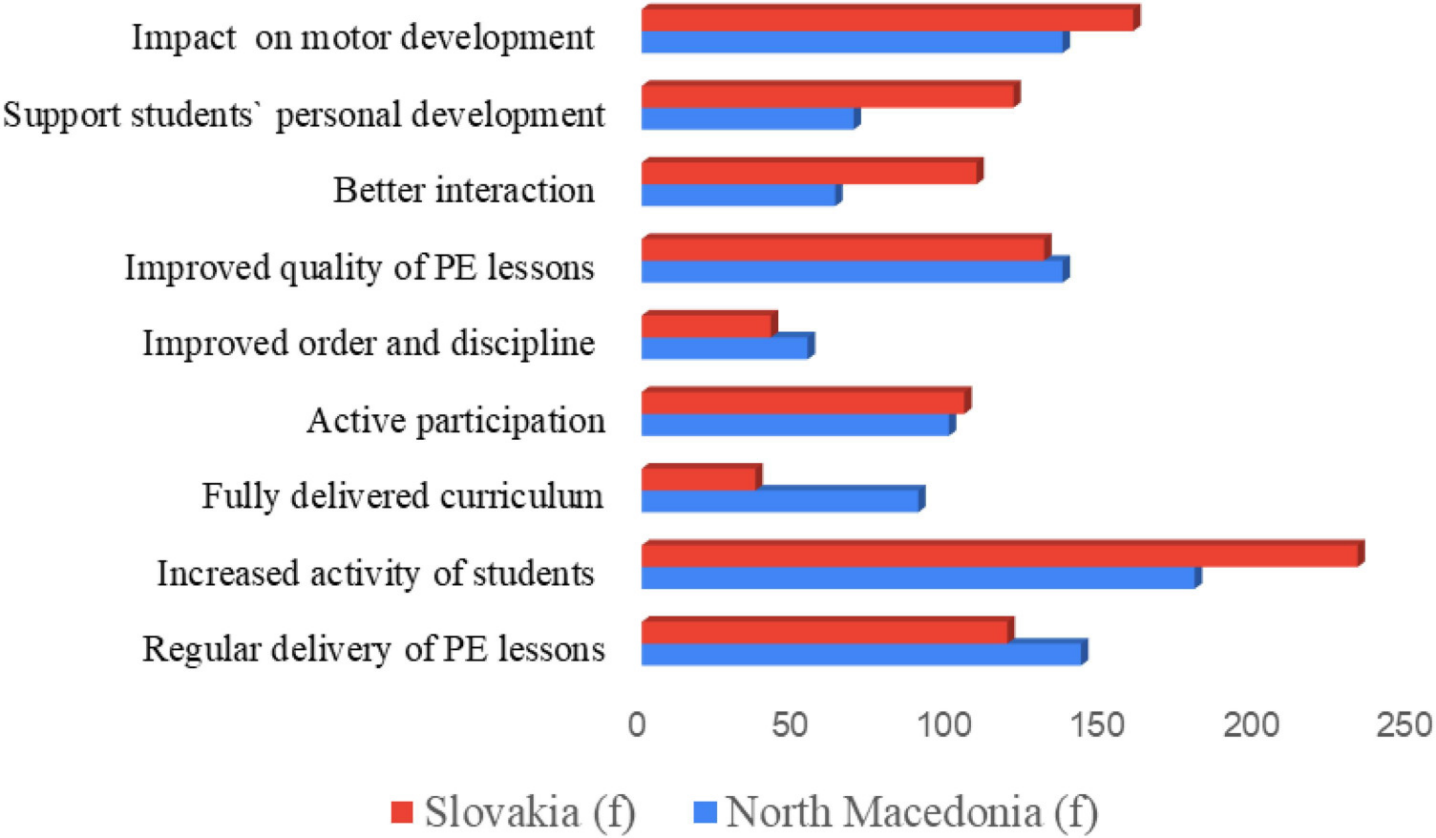
Benefits of tandem teaching perceived by generalist teachers in Slovakia and north Macedonia (frequencies).

Analyzing the frequencies (*f*) of the selections (Table 13, Figure 1), the findings revealed distinct national priorities. Among the Slovak teachers (*N* = 314), the top three reported benefits were: Increased activity of students during PE classes (*f* = 233), followed by the Possibility for systematic influence on motor development and monitoring children's achievements (*f* = 160), and finally, Improved quality in delivery of PE lessons and fulfillment of lesson goals (*f* = 131). For North Macedonian teachers (*N* = 304), the ranking was slightly different: the top benefit was also Increased activity of students during PE classes (*f* = 180), followed by the organizational benefit of PE classes being delivered regularly (no skipping PE lessons) (*f* = 143). The third highest rank was shared by two benefits, both with a frequency of *f* = 137: Improved quality in delivery of PE lessons and fulfillment of lesson goals and the Possibility for systematic influence on motor development and monitoring achievements.

**Table 13:** Generalist teachers' perception of tandem teaching benefits: frequency and rank order of the three most selected benefits by country.

Benefits from tandem teaching	North Macedonia (f)	Slovakia (f)	Ranking
PE classes are delivered regularly (no skipping PE lessons)	143	119	Ranked 2 in North Macedonia
Increased activity of students during PE classes	180	233	Top benefit in both countries
The contents from PE curriculum are fully delivered	90	37	Higher priority in North Macedonia
Possibility for increased attention to each student during PE classes	100	105	Mid-ranking in both countries
Improved order and discipline during PE classes	54	42	Lower priority in both countries
Improved quality in delivery of PE lessons and fulfillment of the goals of the lesson,	137	131	Top 3 in both countries
Possibility for better interaction with all students	63	109	Higher priority in Slovakia
Possibility to support students` personal development	69	121	Moderate ranking, more emphasized in Slovakia
Possibility for systematic influence on motor development and monitoring the achievements of children	137	160	Top 2 in Slovakia; Top 3 in North Macedonia

The most frequently cited benefits across both groups consistently included increased student activity, improved lesson quality, and the systematic influence on motor development. However, a key difference emerged in that North Macedonian teachers prioritized the benefit of regular class delivery (Rank 2), while Slovak teachers gave greater emphasis to the systematic influence on motor development. Conversely, benefits related to classroom management (e.g., improved order and discipline) were consistently ranked lower by both cohorts (*f* = 54 in North Macedonia, *f* = 42 in Slovakia). Overall, the findings suggest that teachers value tandem teaching primarily for its direct impact on student engagement and activity, lesson quality, and the systematic monitoring of learning outcomes, while supportive or organizational benefits vary by national context.

**Figure 2:**
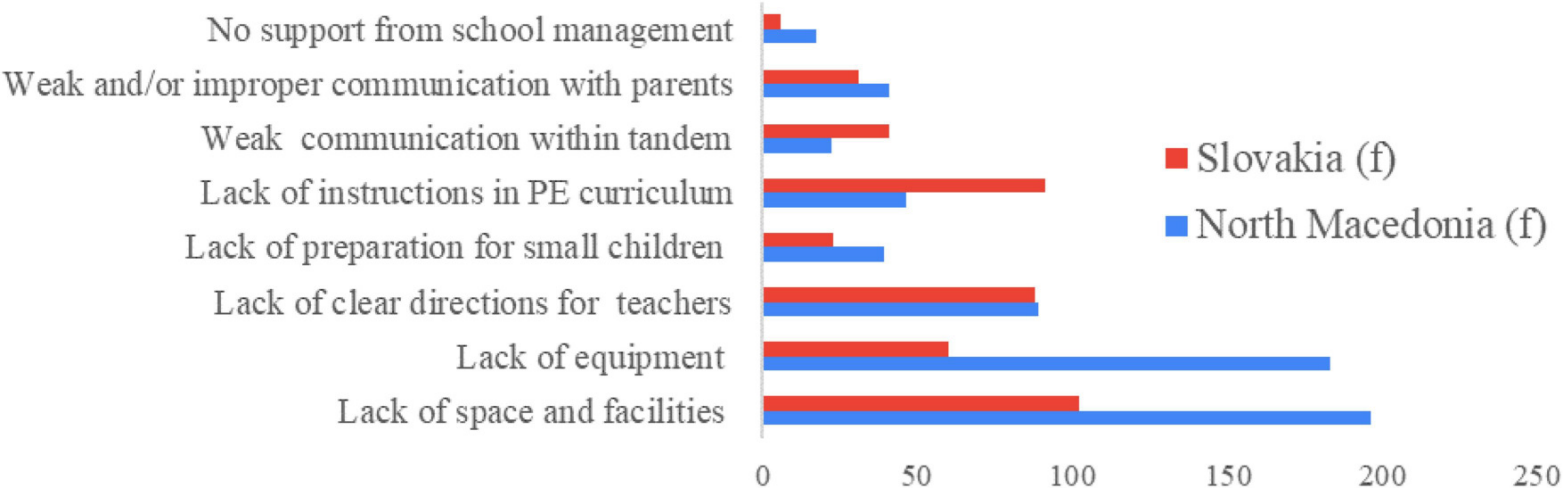
Challenges of tandem teaching perceived by generalist teachers in Slovakia and north Macedonia (frequencies).

Teachers were presented with a list of eight challenges related to resources, competences, communication, and support, and were given an open-ended option. The frequency analysis (*f*) of the selected challenges is presented in Table 14 as well as in Figure 2 for better visualisation. The findings indicate that resource constraints constitute the most significant barrier to effective tandem teaching in both countries. The overwhelming top-ranked challenge was the “lack of space and facilities for PE classes” (*f* = 196 in North Macedonia; *f* = 102 in Slovakia), immediately followed by the “lack of equipment and especially age-appropriate equipment” (*f* = 183 in North Macedonia; *f* = 60 in Slovakia). The substantial difference in these frequencies highlights that resource constraints are a far more prominent and severe challenge in North Macedonia compared to Slovakia, likely reflecting structural differences in school infrastructure or access to dedicated PE facilities. The third most frequently cited challenge in both countries was the “Lack of clear directions for tasks and responsibilities of teachers in the tandem” (*f* = 89 in North Macedonia; *f* = 88 in Slovakia), emphasizing a shared organizational deficiency across both models regarding the need for well-defined tandem roles.

**Table 14:** Generalist teachers' perception of tandem teaching challenges: frequency and rank order of the three most selected challenges by country.

Challenges during tandem teaching	North Macedonia (f)	Slovakia (f)	Ranking
Lack of space and facilities for PE classes	196	102	Top challenge in both countries; more severe in North Macedonia
Lack of equipment and especially age-appropriate equipment for PE classes.	183	60	Top 2 challenge in both countries; more severe in North Macedonia
Lack of clear directions for tasks and responsibilities of teachers in the tandem.	89	88	Top 3 challenge in both countries
Teachers in the tandem are not prepared enough to work with children at early—school age	39	23	Moderate challenge
Lack of clear instructions in PE curriculum for delivery of PE content	46	91	More emphasized in Slovakia
Weak and/or improper communication between the two teachers in the tandem or other colleagues in the group	22	41	Moderate challenge
Weak and/or improper communication with parents	41	31	Low-to-moderate challenge
The Management Of The School Does Not Understand The Needs For Functioning Of The Tandem	17	6	Lowest Priority Challenge

The remaining challenges, while less prominent overall, reflected variations rooted in the national contexts. For instance, the challenge of “Lack of clear instructions in PE curriculum for delivery of PE content” was reported with significantly higher frequency in Slovakia (*f* = 91) than in North Macedonia (*f* = 46), suggesting a greater need for subject-specific curriculum support for the Slovak model. Conversely, issues regarding weak or improper communication, either between teachers (*f* = 22–41) or with parents (*f* = 31–41), were reported with low-to-moderate frequencies, suggesting they are relevant but not widespread challenges. Furthermore, the challenge of “The management of the school does not understand the needs for functioning of the tandem” was reported least often (*f* = 17 in North Macedonia; *f* = 6 in Slovakia), indicating that administrative support is generally perceived as adequate.

In summary, structural factors, specifically resource constraints (space and equipment), represent the most significant overall challenges, particularly dominating the findings in North Macedonia. The lack of clear guidance for roles, responsibilities, and curriculum delivery represents a moderately challenging shared organizational barrier in both countries. Overall, structural and organizational factors appear to be the main obstacles to effective tandem PE teaching, while relational and administrative challenges are secondary.

### 3.5 Generalist teachers' suggestions for tandem teaching improvement

The analysis of the open-ended question related to suggestions for improving teaching quality is presented in Table 15. Out of the total sample of 618 participants, 90 teachers from Slovakia and 110 teachers from North Macedonia provided suggestions for topics they consider both interesting and useful.

**Table 15:** Generalist Teachers' Suggestions for Tandem Teaching Improvement: Thematic Categories and Frequency of Responses by Country.

	Topic	Content
North Macedonia	Collaboration between teachers in the tandem	Need for better communication and collaboration with the other teacher in the tandem, particularly underling the necessity of having clearly defined roles and responsibilities with the tandem. “We need a deeper collaboration with the other teacher in the tandem in all areas of work” “Improved communication between teachers and discussion for real problems” “We need clear instructions for our role in the tandem”
	Investment in facilities and equipment	Majority of teachers underline the poor spatial facilities and lack of equipment as aspect that should be improved. They underline the need of new sport halls, regular access to current sport facilities, access to outdoor facilities, equipment appropriates for childrens age “we need new sport hall only for primary school students” “more equipment appropriate for student age is needed” “we need changing rooms for primary school students”
	Clear directions for tasks and responsibilities within the tandem	Teachers underline the need of clear guidance and instructions for tasks and responsibilities of the teachers within the tandem. Few areas for improvement are recognized. They are following: access in the e—journal, evaluation of the students, planning etc.
	Work with students	Teachers identified a need for intervention in the aspect related to students, particularly their needs, interests and communication with students. Following are some of identified issues and suggestions for changes: “Tandem teaching focuses mainly on movement development. This approach puts aside students creative abilities, music and art skills. These students are somehow discriminated, compared to others that are sport gifted” “Mixing students from 1st—4th grade with older students from 6th to 9th grade for purposes of tandem teaching is sometimes problematic for younger ones. This should be changed”
	Trainings for professional development	Emphasized need of additional trainings in several areas related to tandem teaching (work in the tandem, delegation of tasks and responsibilities, inclusion in PE, classroom management and socio—emotional support for students, cross -subject links and holistic learning through PE etc. “We need more trainings on different topics” “We need trainings how to work together in the tandem”
Slovakia	Policy and Time Allocation	Teachers emphasized that the most critical systemic failure is the lack of dedicated time for genuine co-planning. They frequently suggested that tandem teaching cannot operate effectively without administrative support for scheduling: “The most important thing is to formally allocate non-contact hours specifically for joint lesson preparation and evaluation. Without this, its just improvisation.” “We need protected time outside of the regular teaching load to sit down and integrate the lesson content."
	Systemic Support and Recognition	Recommendations highlighted the need for the state or school administration to formally recognize the added complexity of collaborative teaching: “Tandem teaching should be financially compensated for both teachers. It is more work than teaching alone.” “It is necessary to introduce clear guidelines from the Ministry of Education that define the specific responsibilities and evaluation criteria for both teachers.” “There should be clear roles regarding who is responsible for documentation and grading, as this often leads to friction.”
	Professional Alignment and Training	Teachers recognized that fixing the logistical issues must be complemented by improvements in collaborative skills and philosophical coherence. They suggested that initial training is insufficient for collaboration: “Better cooperation training is needed before starting, so the specialist and the generalist understand each others roles and teaching philosophies.” “There should be a mandatory meeting at the beginning of the year to align goals and expectations for the teaching partnership.”
	Infrastructure and Equipment	Teachers noted that logistical support for the physical environment remains necessary. Examples included calls for: "Investing in more diverse equipment so we can split the class into two independent groups simultaneously.” "Ensuring the physical teaching space is suitable for two groups and two teachers."

Of the 110 teachers from North Macedonia who shared their perspectives, 18 reported that no change was needed (9 stated “nothing should be changed,” while the other half reported that “everything is OK”). Using qualitative content analyses, five key areas of improvement were identified. These included Investment in facilities and equipment, as the majority of teachers underlined poor spatial facilities and lack of age-appropriate equipment as the primary aspects requiring improvement. Suggestions also focused on Collaboration between teachers in the tandem and the need for Clear directions for tasks and responsibilities within the tandem (covering planning, e-journal access, and evaluation). Finally, teachers identified needs related to Trainings for professional development and improving their Work with students, particularly concerning aligning tandem teaching with students' creative needs and managing mixed grade levels.

The responses from Slovak teachers generated 90 individual coded suggestions, which strongly validated the systemic challenges identified in earlier sections. Suggestions related to Policy and Time Allocation formed the dominant thematic category, accounting for 44 suggestions (48.9%). This finding directly correlates with the primary organizational challenge identified previously, where teachers emphasized that the most critical systemic failure is the lack of dedicated time for genuine co-planning. The second most frequent category focused on Systemic Support and Recognition, garnering 22 suggestions (24.4%). These recommendations centered on the need for official, legislative, and financial acknowledgment of the added complexity of the tandem effort. Professional Alignment and Training represented a significant minority of the responses, with 16 suggestions (17.8%), indicating that teachers recognize that fixing logistical issues must be complemented by improvements in collaborative skills. The least frequent suggestions concerned Infrastructure and Equipment, totaling 8 suggestions (8.9%), noting that logistical support for the physical environment remains necessary.

Presented perspective of teachers from North Macedonia and Slovakia provide valuable insight into teachers’ experiential knowledge and context-specific suggestions for improving the current implementation of tandem teaching in PE. The identified areas for improvement (five in North Macedonia and four in Slovakia) highlight recurring concerns and perceived support needs among participating teachers. Considering that this question was answered by only a subset of the total sample of teachers, the identified areas for improvement should be interpreted as illustrative and exploratory, offering meaningful directions for practice and future research, rather than as representative of the entire study sample or the wider population of teachers in tandem teaching.

## 4 Discussion

### 4.1 Overall perception and implementation quality

The fundamental concept of tandem teaching is strongly endorsed by generalist teachers in both Slovakia and North Macedonia, with overall positive mean ratings. This collaboration is a direct response to the systemic challenges in primary PE delivery, where the focus is on achieving Quality Physical Education (QPE), which is a ‘planned, progressive, inclusive learning experience’ crucial for developing physical literacy (36, 37). This model is specifically recognized as a means to address the inadequate preparedness of generalist teachers for delivering QPE (29, 58). These limitations are part of a global trend, as nearly 57% of primary school PE teachers worldwide lack specialized training, highlighting a systemic deficit in “human capital” for QPE delivery (37). These limitations stem largely from inadequate pre-service training, including insufficient opportunities for practical PE teaching experience and the perceived low status of PE in initial teacher education, which often leads to generalist teachers lacking the confidence and specialized skills required for effective PE delivery (18, 21, 38). The teacher is considered the paramount factor in the effective delivery of QPE (37), making tandem teaching a strategy to enhance teacher competence in the subject (39). The term “tandem,” defined as “*two people or pieces of equipment that work together*” (57), captures the essence of this collaborative approach, which is fundamentally defined by “*two or more professionals delivering substantive instruction to a diverse group of students in a single physical space*” [(1), p. 1]. However, it is important to contextualize the high overall positive mean rating in Slovakia (M = 4.35) by noting a potential selection bias. The Slovak sample consists of teachers from schools that voluntarily opted into the “Coaches in Schools” project, which may artificially inflate the positive perception compared to a system-wide, mandatory evaluation. While the idea is popular, the institutional support structures for implementation vary significantly. The structural distinctions between the two national programs are clear: the Slovak model utilizes rotational sports coaches and co-teaches only 1 of 3 weekly lessons, whereas the North Macedonian model employs consistent PE teachers for all 3 weekly lessons.

The data shows that generalist teachers in North Macedonia perceive the program as being more systematic and better supported than their counterparts in Slovakia. North Macedonian generalists reported higher satisfaction levels regarding teacher support, systematic implementation, and clear curricular guidelines. This finding is counterintuitive given the broader scope and demands of the Macedonian program (whole-year commitment, all lessons). We hypothesize that the broader scope and whole-year commitment in North Macedonia necessitated a more robust, centrally structured, and mandatory institutional roll-out, which fostered greater policy fidelity and institutionalization of change compared to the Slovak rotational, limited-scope model. Crucially, the long-term, consistent partnership structure in North Macedonia appears to be a key factor in enhancing the generalist teachers' preparedness and professional growth in QPE. This model operates as a form of “Co-Professionalization,” strategically leveraging the specialist's expertise to build the capacity of the non-specialist and thereby upskilling the workforce without lowering the pedagogical standard (29). The positive sentiment in the Slovak model is also robust, with a three-year analysis of the “Coaches in Schools” project demonstrating consistently high teacher satisfaction across multiple years. Generalist teachers in Slovakia overwhelmingly supported the long-term, weekly involvement of the external providers, with “Yes” responses consistently averaging around 80% and increasing to 84% in the final year (40). Conversely, the lack of clear role expectations was perceived as a universally shared problem across both systems, indicating that while institutional structures differ, the fundamental ambiguity in dividing labor remains a common challenge.

### 4.2 Structural divergence in task involvement and responsibility

The quantitative results, focusing on task involvement and responsibility division, reveal highly statistically significant structural differences in how tandem teaching is implemented in the two national contexts. The Chi-Square test established a highly significant association between the country (Slovakia vs. North Macedonia) and the generalist teacher's reported level of involvement and responsibility for every task analyzed (*p* < 0.001 for all tasks). This revealed that while the tandem concept in North Macedonia is characterized by a high degree of collaboration and shared responsibility, the roles in Slovakia exhibit pronounced structural specialization, reflecting a clear vertical division of labor in both pedagogical and administrative duties. The findings suggest that the divergence between the North Macedonian and Slovak models is primarily structural, rooted in policy-driven role definitions rather than fundamental pedagogical differences. However, this interpretation must be treated with caution. As this study is based on teacher perceptions, direct observational evidence of actual teaching practice would be required to confirm whether these structural differences translate into distinct pedagogical shifts in the gym.

This specialization in the Slovak context closely aligns with non-collaborative co-teaching structures, such as the “One Teach, One Assist” (1) or “One Teach, One Observe” model (10)—approaches frequently cited as the most prevalent in general classroom settings (40). The preference for “One Teach, One Assist” is widely observed because it requires less joint planning (40). Although this reduced effort can consequentially limit the collaborative effort crucial for fostering student success, the effectiveness of true tandem teaching is fundamentally rooted in a continuous cycle of co-planning, co-teaching, co-assessing, and co-reflecting (2, 3). This continuous cycle is the foundation of interprofessional collaboration, which is based on a shared commitment to mutual goals, joint responsibility, and mutual accountability (41, 42). The observed deviation from this collaborative cycle highlights that the Slovak generalist teacher's role is substantially diminished in content-focused domains and primarily channeled into in-class support and management.

The most significant structural divergence was specifically identified in conceptual planning tasks. In the *Planning of the content of the PE lesson*, a striking 31.5% of Slovak generalist teachers reported being “not involved at all” (compared to only 5.9% in North Macedonia). This difference was statistically confirmed as having a large effect size, strongly indicating a vertical specialization structure where the PE specialist predominantly holds the content expertise and lesson design authority. Similarly, for the *Selection of organizational forms for PE lessons*, 32.5% of Slovak teachers were “not involved at all” (vs. 9.2% in North Macedonia), a difference with a large effect size. This pattern of specialization was also reflected in the *Selection of the equipment needed for PE lessons*, where 33.1% of Slovak teachers reported “not involved at all” vs. 10.2% in North Macedonia.

In contrast to their minimal input on content, the Slovak generalist teacher primarily provides critical in-class support, showing high engagement in tasks like *Maintaining the socio-emotional climate* and *Keeping order and discipline*. This supportive function aligns with the definition of the “One Teach, One Assist” model. Furthermore, the Slovak generalist teacher is most frequently completely involved (50.6%) in *Communication with parents*, suggesting this non-instructional duty is distinctly separated from the coaching role. The Slovak tandem, therefore, appears to prioritize the transfer of content and technical knowledge (driven by the specialist) over mutual co-design of lessons, with the generalist fulfilling a supportive and administrative function.

Conversely, the North Macedonian data, showing much higher generalist involvement across the entire planning spectrum and a greater propensity for working “together with the colleague” in execution tasks, points towards a model closer to “Parallel Teaching” or “Teaming” (1, 10), suggesting a more horizontal, shared approach to instructional duties. This data, which describes the collaborative work and shared responsibility in North Macedonia, is based on the generalist teacher's self-perception and should be interpreted as such. The strong data for shared involvement is particularly characteristic of Teaming. In all core planning and content tasks, the North Macedonian generalist teacher's most frequent response was “I am completely involved” (ranging from 37.5% to 41.8%), reflecting a high sense of individual responsibility that rivals that of the specialist. Furthermore, the combined percentage of those reporting “I am completely involved” or “We are working together” is substantially higher in North Macedonia for critical tasks. For *Choosing activities and content*, the combined rate reached 71.4% in North Macedonia (38.8% completely involved +32.6% working together), in stark contrast to only 23.3% in Slovakia. A similar pattern of joint ownership was observed in *Preparing students for PE lessons*, where North Macedonia reported 73.4% combined involvement (41.8% completely involved +31.6% working together), compared to 54.1% in Slovakia. This greater overall involvement and collaborative tendency suggests a more fluid, co-instructional partnership where the generalist teacher is an integral and equally responsible part of lesson design and execution, a hallmark of shared tandem teaching models. This high degree of horizontal collaboration—particularly the shared ownership of content design—is essential for promoting pedagogical competence and fostering high-fidelity integrated instruction, aligning with best practices for sustained collaborative models (43). The adoption of such rigidly delineated role divisions, particularly in Slovakia, may be partially attributed to systemic constraints that inhibit partnership stability and collaborative fidelity. A key factor is the potential for a rotational model where teacher pairings are frequently altered by school leadership. This lack of consistency prevents partnerships from developing the sustained rapport necessary for integrating more complex, collaborative instructional practices (17). Similarly, the feasibility of sustained co-teaching is compromised if one educator is routinely reassigned to other duties (16). As Qualls et al. (16) assert, inconsistency in key partnership variables—such as content understanding, instructional expertise, or overall experience—precludes the true establishment of a functional collaborative system. It is therefore posited that the observed degree of collaboration and shared responsibility is directly dependent on the stability and quality of the tandem teaching practice (43, 44).

Furthermore, the findings reflect a major and recurrent limitation in the literature: the widespread deficit in professional preparation for tandem teaching. Many educators lack formal training in collaborative practices, a skill increasingly viewed as essential for 21st-century education. This problem is compounded by the insufficiency of dedicated planning time (11), which is consistently cited as a primary obstacle that directly hinders shared planning (45). Specific program variables may also contribute to the observed specialization. For instance, the involvement of an external coach who participates in only a limited number of lessons may lead to a reduced sense of ownership or responsibility for the overall effectiveness of the educational process. Furthermore, the low volume of instruction (e.g., one out of three weekly PE lessons being tandem taught) may structurally constrain the investment of time and resources required for robust co-planning and shared responsibility.

### 4.3 Benefits, challenges and suggestions

The final analysis of benefits, challenges, and suggested improvements strongly indicates that while both tandem models are effective in achieving core pedagogical goals, they are fundamentally constrained by structural and administrative factors. Tandem teaching is widely considered a valuable professional development strategy (46, 59), especially for its capacity to facilitate informal learning that enhances teachers' knowledge, skills, and attitudes (7, 47). It is also known to improve the quality of PE by combining the pedagogical skills of generalist teachers with the specialized expertise of external providers, which in turn enhances teacher confidence and increases student engagement (29, 60). The theoretical foundation of this approach aligns with the socio-constructivist perspective of learning, proposing that teachers who collaborate are more effective (7, 61).

The results reveal that while both national models prioritize different structural outcomes—North Macedonian teachers highly valued the organizational benefit of regular class delivery, while Slovak teachers emphasized the *Possibility for systematic influence on motor development*—the paramount benefit in both countries was Increased student activity. This finding is critical as it suggests a universal validation of the tandem model's primary pedagogical objective: ensuring active student engagement. This is strongly supported by the three-year data from Slovakia, where teachers consistently rated children's active engagement as “Excellent” by a significant majority (87%–90%) and rated the physical activities as having “Maximum” attractiveness (82%–88%) (40). Furthermore, student perception data provides additional evidence for this benefit: in the Slovak context, students in the tandem teaching group rated their coaches significantly higher in several instructional aspects compared to the control group, and the intervention was associated with a slightly increased propensity for PE as a favorite subject (30). This positive impact on student enjoyment and motivation is vital, as instructor behavior is a key determinant of children's enjoyment of PE (48). For students, the model provides a richer learning environment with diverse perspectives and teaching styles and allows for more personalized attention and quicker assistance (7, 8, 49), thereby improving group discipline and facilitating continuous progress monitoring (50). This consensus establishes that, despite structural and organizational failures, the fundamental goal of increasing physical activity—a core metric for modern physical literacy policies—is being successfully met.

The analysis of challenges indicates that resource constraints constitute the most significant barrier to effective tandem teaching. The overwhelming frequency of challenges related to the lack of space/facilities and equipment in North Macedonia highlights a severe systemic barrier rooted in infrastructural investment that outpaced policy rollout. This challenge is confirmed as a widespread structural barrier globally; the under-prioritization of PE results in 63.8% of countries allocating less than 2% of their national education budgets to PE, leading directly to underfunded facilities and resources (37). Crucially, a shared organizational deficiency across both models is the high ranking of the challenge related to lack of clear directions for tasks and responsibilities. This widespread organizational ambiguity demonstrates a systemic failure across national policies to formally define the division of labor, leading to reliance on ad-hoc, informal agreements. This shared deficit is compounded by another key finding: a significant deficiency in generalist teachers' preparedness in both countries for working with children with diverse learning needs (29), a common challenge linked to generalists' lack of inclusive teaching strategies (51–53).

This shared deficit is immediately addressed by the teachers’ open-ended suggestions, which fundamentally request policy remediation over relational training. The teachers' requests for non-contact time and policy support are substantiated by global trends showing that opportunities for continuous professional development (CPD) in PE are scarce due to budgetary constraints and lack of time, representing a systemic under-investment in staff training (37). The teachers' consensus is that the tandem model cannot succeed based solely on teacher goodwill; it requires formal administrative recognition and restructuring. A primary obstacle identified in the literature is the lack of sufficient time for co-teachers to collaborate, often due to heavy workloads (11, 45). This is most evident in the finding that nearly half of all Slovak suggestions focused on *Policy and Time Allocation* (48.9%). This directly signals that their short-term, rotational model fails because it does not budget the non-contact time essential for genuine co-planning and evaluation. Further challenges noted in the Slovak context included a broader concern in the literature regarding the risk of diminishing the role of the generalist teacher in PE due to outsourcing (21). This risk is often fueled by the potential for external providers (coaches) to lack comprehensive knowledge of the school curriculum, adequate pedagogical skills, or familiarity with individual students, a crucial distinction compared to certified PE specialists (54–56). However, the Slovak “Coaches in Schools” project, which emphasizes co-teaching and includes a mandatory 3-day training program for coaches (40), appears to successfully mitigate this risk by actively involving teachers in the instruction process. The structural nature of these requests aligns with recommendations that policymakers should formally recognize and support collaborative teaching as a viable, job-embedded CPD strategy (29, 59). These suggestions collectively establish a clear sequence for improvement: structural issues (resources/facilities) must be solved first, followed immediately by mandated policy changes (defining roles and guaranteeing time for planning).

In synthesizing these requests, a unified theme emerges: the urgent need for the Systemic Formalization of the Tandem Model. The recommendations highlight that fixing the challenges requires moving beyond informal, voluntary cooperation toward mandated policy support. This points to the firm conclusion that tandem teaching is currently under-resourced and under-formalized across both national systems, and its successful future depends on its integration into the school's formal administrative and financial structure. Specifically, policy must mandate long-term partnerships rather than short-term rotations for the tandem system to develop and flourish. Furthermore, to address the systemic deficit in inclusive education, pedagogical faculties and Continuing Professional Development (CPD) programs must place greater emphasis on inclusive teaching strategies during both pre-service training and ongoing development. Crucially, the long-term effectiveness of the program is contingent upon addressing the lack of standardized assessment of student outcomes in areas such as motor skills, physical fitness, and PE knowledge (29).

### 4.4 The influence of contextual factors: teacher experience and program duration

A critical lens for interpreting the comparative differences between the Slovakian and North Macedonian teachers is the significant sample heterogeneity across key demographic variables: professional experience and grade-level involvement (Tables 2–4). These are not merely statistical limitations, but powerful contextual factors that may explain the observed differences in teacher perceptions.

The samples differ significantly in their exposure to teaching and collaboration. Firstly, the Slovakian sample is disproportionately composed of veteran teachers (59.2% with 21+ years experience), whereas the North Macedonian sample is younger (45.7% with 10 or fewer years of experience), (see Table 2). This veteran presence in SK may influence their perceptions of professional growth, potentially leading to greater resistance or criticality regarding the new model compared to the younger, possibly more adaptable NM group. Secondly, the maturity of the program within each country is highly varied. The majority of Slovakian participants (72.6%) reported only 1–2 years of tandem experience, while the majority of North Macedonian participants (65.4%) reported 3–5 years (see Table 3). This difference is crucial because NM teachers have had more time to move beyond the initial, often difficult, “forming” stages of collaboration, allowing for the resolution of initial logistical and interpersonal conflicts. Their perceptions are likely shaped by routinized, established collaborative practice, potentially leading to higher reported satisfaction and lower reports of minor challenges. In contrast, SK teachers, being newer to the model, are more likely to be reporting on the steeper initial learning curve, where planning is inefficient and roles are still being negotiated (44).

In addition, the pedagogical demands placed on the collaborating teachers are dictated by the grade level, which also differs markedly between the countries (see Table 4). The SK sample is highly concentrated in Grades 1 and 2 (71.1%). Collaboration in these early grades typically focuses on classroom and behavior management, safety, and the delivery of fundamental motor skills. The generalist teacher's primary benefit from the coach may be receiving assistance with these high-intensity logistical and safety concerns. On the contrary, the NM sample is more involved in upper primary grades (Grade 4–5) and, notably, a much higher proportion are engaged in Multi-Grade Teaching (28.3%). Collaboration in upper grades requires a focus on content knowledge transfer for specific sports and curriculum differentiation for a more diverse student skillset. The high incidence of multi-grade teaching in NM presents unique, systemic challenges for planning and implementation, requiring a much higher degree of flexible co-teaching models and strategic lesson design, which could influence reported planning challenges and perceptions of collaborative effectiveness.

These contextual variations suggest that differences in perceptions may reflect the maturity of the program and the specific pedagogical demands of the age group being taught, rather than solely the superiority of the coach-led vs. the PE teacher-led model.

### 4.5 Study limitations

The study's findings must be interpreted in light of several methodological limitations, which primarily relate to the research design and the instrument used for data collection.

- Instrument Development and Formal Standardization: A primary limitation is the use of a non-formally standardized instrument. Although extensive measures were taken to ensure face and content validity—including expert consultation, translation, and pilot testing—the questionnaire was specifically developed for this context and was not subjected to full psychometric validation and norming procedures prior to this data collection. This necessitates caution when comparing the absolute magnitudes of the mean values across different sub-sections of the instrument.
- Sample Heterogeneity: The non-homogeneous nature of the two national samples regarding general working experience, specific tandem teaching experience, and the grade levels served (SK focused on Grade 1–2; NM on upper/multi-grade levels) introduces a significant uncontrolled variable. This prevents direct causal attribution of observed differences solely to the model type and must be carefully considered when interpreting the comparative results.
- Selection Bias in the Slovak Sample: The recruitment method for the Slovakian cohort, drawn exclusively from schools that voluntarily signed up for the “Coaches in Schools” project, introduces a significant selection bias. Teachers participating in the survey were pre-selected based on their school's willingness to engage in the tandem model, suggesting they likely possess a higher degree of motivation, enthusiasm, or alignment with the program's goals compared to the general population of Slovak primary teachers. This pre-selection may have influenced their perceptions to be artificially more positive regarding the overall concept (Section 3.1) and general implementation quality (Section 4.1).
- Cross-Sectional Design and Causal Inference: The reliance on a cross-sectional design captures teachers' perceptions and experiences at a single point in time. This design prevents the establishment of causal relationships between the structural differences and the reported outcomes. The perceptions reported may be influenced by transient factors or specific partnership histories that the study design cannot account for.
- Self-Report and Social Desirability Bias: The data relies entirely on self-report measures, which introduces the risk of social desirability bias. Teachers may tend to over-report collaborative or professionally desirable behaviors, particularly regarding task involvement and shared responsibility. This potential bias may have artificially inflated the reported levels of cooperation and obscured relational conflicts.
- Missing Partner Perspective (Dyadic Limitation): A critical limitation is the exclusion of the tandem partner's perspective. The study only captures the viewpoint of the generalist teacher, providing an incomplete, single-sided picture of the collaborative dynamic. Contextual Specificity: While the comparative design is a strength, the conclusions are highly context-dependent. The findings are specific to two unique tandem models operating under distinct national policies. The conclusions regarding implementation challenges and best practices are therefore not directly transferable to different co-teaching settings or countries with alternative policy structures without further validation.

Despite these constraints, this study provides a valuable baseline for understanding national differences in tandem teaching. Future research should aim to triangulate these findings through direct classroom observations, documentary analysis of curricular policies, and qualitative interviews with both generalist teachers and PE specialists or coaches to provide a more holistic understanding of tandem teaching dynamics.

## 5 Conclusions

This study, involving 618 generalist teachers across Slovakia (*n* = 314) and North Macedonia (*n* = 304), provides a robust comparative foundation for understanding the operational realities of tandem teaching in primary PE. The findings confirm that while the overall concept of collaborative teaching is universally embraced and positively perceived, the structural implementation models result in fundamentally different professional roles and responsibilities for generalist teachers.

### 5.1 Scientific contributions

Based on the comparative analysis, this research offers three primary scientific contributions to the field of collaborative pedagogy:

- The study demonstrates that while national policy significantly diverges in structural areas like planning and administration—where the Slovak “external coach” model leads to vertical specialization and the North Macedonian “internal specialist” model fosters shared ownership—pedagogical delivery remains remarkably uniform across contexts. This suggests that “on-the-ground” instructional practices in PE are more influenced by shared professional norms than by systemic organizational frameworks.
- The research provides empirical evidence that sustained partnerships, as seen in North Macedonia, significantly increase the depth of collaboration in non-instructional tasks, such as strategic lesson planning, the selection of pedagogical organizational forms, and the systematic preparation of students for physical activity, compared to rotational models.
- The study contributes to implementation theory by demonstrating that collaborative pedagogical reforms have a “threshold of material necessity.” It reveals that, regardless of the excellence of a policy model, systemic success is limited by the physical environment, suggesting that human capital interventions (such as tandem teaching) cannot compensate for infrastructure deficits.

### 5.2 Summary of key findings

- Divergent Role Division: The most pronounced differences between the two models appeared in preparatory responsibilities. While the Slovak model is characterized by a specialized, delegated approach where generalist teachers are largely excluded from content planning, the North Macedonian model fosters a high degree of shared involvement and mutual responsibility.
- Systemic Support Deficit: North Macedonian teachers reported a significantly more structured and better-supported implementation process. This contrast highlights the pedagogical benefits of long-term, comprehensive legislative commitment compared to more flexible or rotational pilot programs.
- Universal Challenges: Despite systemic differences, both contexts identified the positive impact on student activity and lesson quality as the primary benefits. Conversely, both systems suffer from severe structural barriers—most notably, the lack of appropriate facilities and equipment—which remains the most significant hurdle to successful tandem teaching regardless of the national model.

### 5.3 Policy and practice recommendations

To optimize collaborative teaching, policy recommendations include:

- Systemic Formalization and Time Allocation: Policymakers should mandate protected co-planning time to address the constraints that hinder sustained collaboration.
- Clarity of Administrative Framework: Legislation should formally define and codify roles and responsibilities for all tandem partners (addressing the universal “organizational ambiguity”), integrating North Macedonia's shared ownership with clear guidelines for delegation.
- Infrastructure Investment: Significant investment in facilities and equipment is required to address the most prominent logistical barriers identified in both nations.

In conclusion, the optimal tandem teaching model for primary PE would integrate North Macedonia's sustained partnership and shared ownership with Slovakia's clarity in administrative delegation. Finally, it must be noted that these recommendations should be viewed as “improvement hypotheses” that require further evaluation through longitudinal or mixed-methods designs to confirm their long-term impact on educational outcomes.

## Data Availability

The datasets presented in this study can be found in online repositories. The names of the repository/repositories and accession number(s) can be found below: ZENODO: https://doi.org/10.5281/zenodo.17692583.
